# Incidence of glomerulonephritis and non-diabetic end-stage renal disease in a developing middle-east region near armed conflict

**DOI:** 10.1186/s12882-018-1062-7

**Published:** 2018-10-11

**Authors:** Alaa A Ali, Dana A Sharif, Safa E Almukhtar, Kais Hasan Abd, Zana Sidiq M Saleem, Michael D Hughson

**Affiliations:** 1 Department of Pathology, Shorsh General Hospital, Qirga Road, Sulaimaniyah, Kurdistan Iraq; 2 Department of Medicine, Sulaimaniyah University, Sulaimaniyah, Iraq; 3 Hawler University College of Medicine, Erbil, Iraq; 4 Dohuk University, Dohuk, Iraq

**Keywords:** Glomerulonephritis, FSGS, ESRD, Middle-east, Iraq

## Abstract

**Background:**

Estimates of the incidence of glomerulonephritis (GN) and end-stage renal disease (ESRD) in an Iraqi population are compared with the United States (US) and Jordan.

**Methods:**

The study set consist of renal biopsies performed in 2012 and 2013 in the Kurdish provinces of Northern Iraq. The age specific and age standardized incidence of GN was calculated from the 2011 population. ESRD incidence was estimated from Sulaimaniyah dialysis center records of patient’s inititating hemodialysis in 2017.

**Results:**

At an annual biopsy rate of 7.8 per 100,000 persons in the Kurdish region, the number of diagnoses (2 years), the average age of diagnosis, and annual age standardized incidence (ASI)/100,000 for focal segmental glomerulosclerosis (FSGS) was *n* = 135, 27.3 ± 17.6 years, ASI = 1.6; and for all glomerulonephritis (GN) was *n* = 384, 30.4 ± 17.0 years, ASI = 5.1. FSGS represented 35% of GN biopsies, membranous glomerulonephritis 18%, systemic lupus erythematosus 13%, and immunoglobulin A nephropathy 7%. For FSGS and all GN, the peak age of diagnoses was 35–44 years of age with age specific rates declining after age 45. The unadjusted annual ESRD rate was 60 per million with an age specific peak at 55–64 years and a decline after age 65. The assigned cause of ESRD was 23% diabetes, 18% hypertension, and 12% GN with FSGS comprising 41% of biopsy-diagnosed, non-diabetic ESRD.

**Conclusions:**

The regional incidence of ESRD in Northern Iraq is much lower than the crude incidences of 100 and 390 per million for Jordan and the US respectively. This is associated with low renal disease rates in the Iraqi elderly and an apparent major contribution of FSGS to ESRD.

## Background

Chronic kidney disease (CKD) is being diagnosed with increased frequency worldwide [1–3]. Glomerulonephritis (GN) is a major cause of CKD but is estimated to account for only 10% of end-stage renal disease (ESRD) in the West and in the developed countries of Asia where more than 75% of ESRD is attributed to diabetes and hypertension [4, 5]. Many of the larger countries of the Middle-East are unable to collect comprehensive data on kidney disease, and the incidence of CKD and the extent to which GN or diabetes contributes to ESRD are poorly defined [6].

The regional frequencies of different types of CKD are mainly determined by the proportions of diagnostic categories in biopsy series. These studies have shown that IgA nephropathy (IGAN) is the predominant form of CKD in many European and Asian populations but is rare among persons of African ancestry and, when compared to Europe, is relatively uncommon in the Middle-East [7, 8]. An increased frequency in the diagnoses of focal segmental glomerulosclerosis (FSGS) seems to have begun in the early 1980s in the United States (US) and Brazil and in the 1990s in Singapore and India, and FSGS is now the major cause of nephrotic syndrome in many parts of the world [7, 9–12].

In the US and Australia, biopsy registries from well-defined populations have been used to estimate the incidence of specific types of glomerular diseases [13, 14]. In both countries, GN rates composed largely of IGAN and FSGS increased steadily with age, with the relative risk of GN, diabetes, and hypertension all contributing to a growing population of older ESRD patients.

The Middle-East is often considered a homogeneous region with respect to disease [15]. We question this assumption. Conflict has been a part of Iraqi life for nearly 30 years, and epidemiologic record keeping on non-communicable diseases other than cancer is only beginning to receive attention. This current study used a renal biopsy registry from the largely Kurdish population of Northern Iraq to investigate the incidence of the common forms of GN that might lead to ESRD. We also applied the proposals by Anand et al., [15], on the use and initiation of dialysis services as surrogates for prevalence and incidence of ESRD when regional or national data is not available. The findings are compared to Jordan, the adjacent country having a rigorous collection of nationwide data on ESRD, to assess how the findings in Northern Iraq might contribute to a better understanding about the variability of CKD in the region.

## Methods

### Data collection and calculation of age specific glomerulonephritis incidence

Study subjects consisted of patients having renal biopsies performed in the Kurdish region of Northern Iraq between Jan 1 2012 to Dec 31, 2013. This was the 2 year period before the large population dislocations caused by the ISIS conflict that began in March 2014. All biopsies were studied by light microscopy in 18 serial sections using hematoxylin and eosin, periodic acid-Shiff, Masson trichrome, and Jones methenamine stains, and by immunofluorescence microscopy with fluorescein conjugated anti-human IgG, IgM, IgA, C3, C1q, and albumin. Selected cases were sent off-site for electron microscopy. The 2011–2012 United Nations Iraq population data [16] and the 2012 Iraqi Cancer Registry [17] was used for estimates of the Kurdistan population in 5 year age ranges from 0 to 65+ years old. Age specific incidence per 100,000 population was calculated for focal segmental glomerulosclerosis (FSGS), membranous glomerulonephropathy (MGN), systemic lupus erythematosus nephritis (SLE), immunoglobulin A nephropathy (IGAN), and all glomerulonephritis (GN). Minimal change disease (MCD) was not included in all GN, because it generally contributes little to the risk of ESRD.

### Kurdistan, Olmstead County, Minnesota, and Victoria, Australia standardized glomerulonephritis incidence calculations

The Olmstead County and Victoria studies [13, 14] were chosen for comparisons with Kurdistan, because they provided age standardized incidence rates or enough information that standardized incidence rates could be calculated. The United States (US) 2000 Census [18] was used for age adjustments for Kurdistan and Victoria because this was applied to the Olmsted county data.

### Estimates of ESRD incidence in Sulaimaniyah governate; comparison with the kingdom of Jordan

The numbers of patients in 2017 who received dialysis at the regional centers in Sulaimainyah City were tabulated by age. Monthly records were searched for persons dialysed in prior months and for those initiating dialysis. An annual average for all dialysed persons and for persons initiating dialysis was calculated from monthly data. The centers served a catchment area of an estimated 1,310,000 persons having an age distribution similar to that of the rest of the Kurdish region. Crude (unadjusted), age specific, and age adjusted incidence rates were determined. Data from the National Registry of End-Stage Renal Disease of the Kingdom of Jordan was compared with Kurdistan [19].

## Results

### Analysis of the Kurdistan 2012 and 2013 biopsies

Biopsies of 763 native kidneys were received in the 2-year period, 622 of the biopsies were satisfactory for specific diagnoses that are listed in Table 1. The indicated reasons for the biopsies were nephrotic syndrome 52%, proteinuria and hematuria 31%, hematuria without proteinuria 5%, renal insufficiency 7%, and unspecified 1%.

**Table 1:** 2012–2013 renal biopsy diagnoses. Sub-types of diseases are listed

Diagnosis	n	Male	Age (years)	Subtypes (n)
MCD	82	58%	21.5 ± 15.8	IgMN(2), FGGS(4)
FSGS	135	53%	27.3 ± 17.6	Collapsing(2)
MGN	71	46%	37.1 ± 15.0	
SLE	51	16%	30.5 ± 11.8	Class I (3), II(3), III(9), IV(23), V(10), VI(3)
IGAN	28	61%	29.5 ± 17.0	Haas class I(3), II(8), III(7), IV(3), V(3), HSP(4)
Crescentic GN	26	54%	37.1 ± 19.8	Pauci-immune(21); crescentic class(6), mixed class(8), sclerotic class(7). AGBMD(2), ICD(3)
MPGN	12	75%	37.8 ± 20.3	Type 1(11), type2(0), cryoglobulinemia-associated(1)
PSGN	10	80%	18.0 ± 19.5	
Other GN	18	56%	29.9 ± 14.4	MePGN(13), C1qN(2), fibril GN(1), diffuse mesangial sclerosis(1) Obesity-associated glomerulomegaly(1)
CGN-NOS	24	71%	27.2 ± 14.0	
HUS	9	55%	29.0 ± 20.5	D+ (3), APL(1), PP(2)
TIN	46	55%	32.9 ± 20.4	acute pyelonephritis(6)
arteriolosclerosis	52	48%	49.6 ± 15.2	
Amyloidosis AL	2	40%	55.5 ± 0.7	Myeloma(2)
Amyloidosis AA	19	40%	40.2 ± 23.4	RA(4), JRA(3), arthritis(2), SLE (1), FMF(1), asthma(1), bronchiectasis(3), unknown(4).
Myeloma kidney	4	50%	59.3 ± 7.7	
Diabetes	5	40%	47.0 ± 6.4	
Other non-glomerular	28	46%	29.7 ± 14.7	Acute kidney injury(14),basement membrane disease(2) CKD NOS (10), Fabry’s(1), scleroderma(1)
total	622	51%	30.2 ± 18.6	

*Abbreviations*: *MCD* minimal change disease, *FSGS* focal segmental glomerulosclerosis, *MGN* membranous glomerulonephritis (GN), *SLE* lupus nephritis, *IGAN* immunoglobulin A nephropathy, *MPGN* membranoproliferative GN, *PSGN* post-streptococcal GN, *CGN-NOS ESRD* end-stage chronic GN not otherwise specified, *HUS* hemolytic uremic syndrome, *TIN* tubulointerstitial nephritis, *APL* anti-phospholipid syndrome, *PP* postpartum, *C1qN* C1q nephropathy, *MePGN* mesangial proliferative GN (not IGAN or SLE), *fibril GN* fibrillary GN, *IgMN* IgM nephropathy, *FGGS* focal global glomerulosclerosis, *HSP* Henoch-Schoenlein purpura, *AGBMD* antiglomerular basement membrane disease, *ICD* immune-complex disease, *CKD NOS* chronic kidney disease, not otherwise specified, *RA* rheumatoid arthritis, *JRA* juvenile rheumatoid arthritis, *FMF* familial Mediterranean fever

FSGS was the most frequent diagnosis at 22%, followed by MCD at 13%, and MGN at 11%. SLE, IGAN, crescentic GN, and end-stage chronic glomerulonephritis not otherwise specified (CGN-NOS) represented 8, 5, 4 and 4% of the biopsies respectively. CGN-NOS demonstrated globally solidified glomeruli with non-specific immunofluorescence staining and was thought to largely represent late-stage FSGS. Tubulointerstitial nephritis (TIN) comprised 7% of the biopsies, and chronic kidney disease not otherwise specified (CKD-NOS) was found in 10 cases (1.6%) that had interstitial fibrosis and tubular atrophy but with little inflammation and no glomerular disease. GN that included HUS was diagnosed in 384 biopsies. The age of 66% of GN patients fell between 19 to 41 years old with 24% being ≤14 and 9% being ≥55 years old (Table 2). Overall, the sex distribution was nearly equal, but this varied with disease. SLE was predominantly female (84%), and IGAN predominantly male (61%).

**Table 2:** Age and sex distribution of all glomerulonephritis (without minimal change disease) over the 2 year period 2012–2013

2012–2013									
Age (years)	0–4	5–14	15–24	25–34	35–44	45–54	55–64	65+	Total (male)
FSGS	12 7 M	19 9 M	26 16 M	27 15 M	21 8 M	13 8 M	7 6 M	3 3 M	135 (53%)
MGN	1 M	2 2 M	10 5 M	19 9 M	19 6 M	11 6 M	5 1 M	4 3 M	71 (46%)
SLE		4 1 M	12 3 M	16 0 M	12 2 M	6 2 M		1 0 M	51 (16%)
IGAN	1 0 M	6 2 M	4 1 M	7 7 M	4 3 M	3 2 M	2 1 M	1 M	28 (61%)
Crescentic GN		2 1 M	6 3 M	5 1 M	5 4 M	3 1 M	2 1 M	3 3 M	26 (54%)
MPGN			4 3 M	3 2 M		2 1 M	1 M	2 2 M	12 (75%)
PSGN		8 7 M	1 0 M					1 M	10 (80%)
other GN	2 1 M	1 0 M	4 1 M	5 3 M	4 3 M	1 M	1 M		18 (56%)
CGN-NOS ESRD	1 M	4 2 M	3 M	10 9 M	5 3 M			1 M	24 (71%)
HUS		4 3 M	1 0 M	1 0 M	1 0 M		2 2 M		9 (55%)
Total GN	24 10 M	50 27 M	71 33 M	93 46 M	71 29 M	39 21 M	20 13 M	16 14 M	384 (50%)

*Abbreviations*: *M* male, *FSGS* focal segmental glomerulosclerosis, *MGN* membranous glomerulonephritis (GN), *MPGN* membranoproliferative GN, *IGAN* immunoglobulin A nephropathy, *PSGN* post-streptococcal GN, *SLE* lupus nephritis, *CGN ESRD* chronic GN not otherwise specified at end-stage renal disease, *HUS* hemolytic uremic syndrome

End-stage kidney disease (ESKD) was diagnosed in 78 biopsies (Table 3). The indicated reasons for biopsy was nephrotic syndrome and renal failure in 23 cases, unexplained renal failure with proteinuria in 45 cases, and unexplained renal failure alone 10 cases. Our regional criteria for biopsy does not include confirmation of ESRD, and the diagnosis of an end-stage kidney was largely unanticipated and before dependency on renal replacement therapy was recognized. The majority (55.0%) of these biopsies were end-stage GN. Many of these GN biopsies were categorized as CGN-NOS (30.8%) but also included late-stage FSGS with characteristic segmental lesions (7.7%), SLE class VI (3.8%), Hass class V IgAN (3.8%), and sclerotic class pauci-immune GN (9%). TIN consisted of 16.7% and CKD-NOS 9.0% of ESKD biopsies. The combination of CGN-NOS and late-stage FSGS represented 41% of non-diabetic ESKD biopsies.

**Table 3:** Biopsies having histologic features of end-stage kidneys

ESKD diagnosis	biopsies	Biopsies in category	% for category	% for ESKD biopsies
CGN-NOS, end-stage	24	24	NA	30.8
FSGS, end-stage	6	135	4.4	7.7
SLE class VI	3	51	5.9	3.8
IgAN Haas V	3	28	10.7	3.8
Crescentic GN, sclerotic class	7	26	26.9	9.0
Arterionephrosclerosis, end-stage	2	52	3.8	2.6
diabetes	5	5	100.0	6.4
Amyloid AA	6	19	31.2	7.7
Myeloma cast nephropathy	2	4	50	2.5
TIN, end-stage	13	46	28.3	16.7
CKG-NOS, end-stage	7	10	70	9.0
Total	78	400		100.0

*Abbreviations*: *ESKD* end-stage kidney disease, *FSGS* focal segmental glomerulosclerosis, *SLE* lupus nephritis, *IGAN* immunoglobulin A nephropathy, *CGN-NOS* chronic glomerulonephritis not otherwise specified, *GN* glomerulonephritis, *TIN* tubulointerstitial nephritis, *CKD NOS* chronic kidney disease, not otherwise specified

### Kurdistan population and estimates of age specific and age standardized GN incidence

**Fig. 1:**
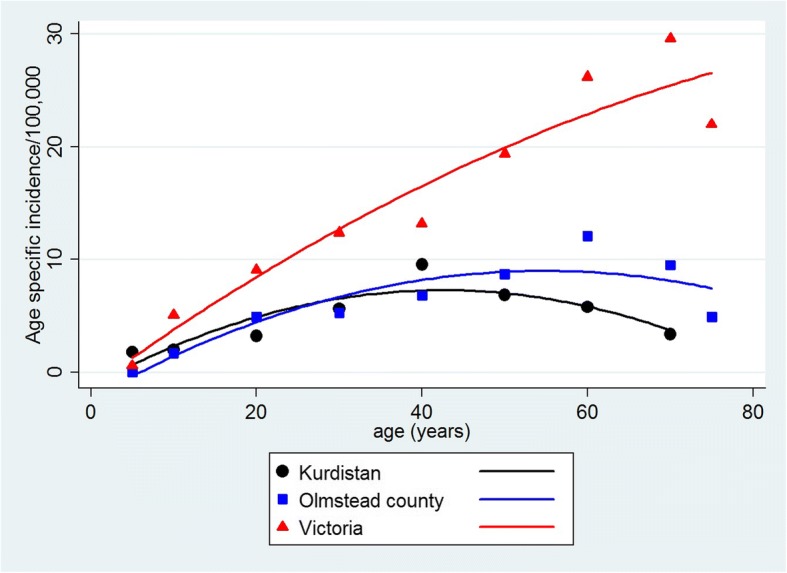
Age specific incidence of all glomerulonephritis in Kurdistan, Olmstead County, Minnesota, and Victoria, Australia. In Kurdistan, all glomerulonephritis reached a plateau at about age 40 and then declined. In Victoria, incidence increased from young to old age with no plateau

The estimated population for Kurdistan in 2011 was 4,900,000 persons. This is a young population characteristic of the Middle East with 61% being under 25 years of age (Table 4). The annual crude biopsy rate in Kurdistan for native kidneys including unsatisfactory specimens for the years 2012–2013 was 7.8 per 100,000 (381 biopsies/4,900,000 persons). The age specific incidence for FSGS, MGN, SLE, IgAN, and all GN are shown in Table 5 together with the total US 2000 age standardized incidence for each diagnosis. GN was rare before 15 years of age, and the highest age specific incidence occurred between 35 and 44 years of age in all diagnostic categories. Thereafter, age specific incidence declined for all disease categories and after age 65 returned to rates seen at 15–24 years old (Fig. 1).

**Table 4:** Population of Kurdistan in 2011 from WHO Population Division estimates

Age	Female	Male	Total
0–4	325,581	345,720	671,301
5–14	624,789	619,811	1,244,600
15–24	541,195	556,405	1,097,600
25–34	413,249	409,953	823,202
35–44	185,622	186,780	372,402
45–54	128,174	156,126	284,300
55–64	84,721	86,779	171,500
65+	117,384	117,816	235,200
total	2,420,715	2,479,390	4,900,105

**Table 5:** Kurdistan, Iraq. Age specific and age standardized (total rate) annual incidence per 100,000 population for FSGS, MGN, SLE, and all GN for the years 2012–2013

Age (years)	FSGS	MGN	SLE	IGAN	All GN
0–4	1.4	0.1		0.1	1.8
5–14	0.8	0.1	0.2	0.2	2.0
15–24	1.2	0.5	0.6	0.2	3.2
25–34	1.6	1.2	1.0	0.4	5.7
35–44	2.8	2.6	1.6	0.5	9.5
45–54	2.3	1.9	1.1	0.5	6.9
55–64	2.0	1.5		0.6	5.9
65+	0.6	0.9	0.2	0.2	3.4
Total rate^a^	1.6	1.2	0.7	0.4	5.1

^a^The total rate is adjusted to the 2000 US standard population

Compared to other disease categories, FSGS had higher age specific incidences from childhood to 64 years old and a higher total age adjusted incidence. The peak age specific incidence for the diagnosis of FSGS occurred at 35–44 years of age when it was 2.8/100,000 compared to 2.6/100,000 for MGN. The age standardized incidence of SLE for females was 1.07/100,000 compared to 0.2/100,000 for males. For IGAN the age standardized incidence for males was 0.4/100/000 compared to 0.2/100,000 for females.

### Comparisons of GN incidence in Kurdistan, Olmstead County, Minnesota, and Victoria, Australia

Table 6 compares the incidence of all GN, FSGS, IGAN, SLE, MGN, and ESRD in Kurdistan (2012–2013), Olmsted County (1994–2003), and Victoria (1995 and 1997) with the proportion of diabetics entering ESRD programs. The differences in all GN between Kurdistan, Olmsted County, and Victoria were largely due to the much higher rates of IGAN in Olmstead County and Victoria and the high rate of SLE in Victoria. The standardized rates for MGN and FSGS were somewhat similar for the three regions. Olmstead county had twice the incidence of ESRD as Victoria with 25% more diabetes.

**Table 6:** Comparison of age standardized annual incidence (per 100,000) of all glomerulonephritis, FSGS, IgAN, LN, and MGN in Olmsted County MN (USA); Victoria, Australia; and Kurdistan of Iraq. The ESRD incidence and the percent of diabetic ESRD is provided for each region

Country	All GN	FSGS	IgAN	SLE	MGN	ESRD incidence^a^	% ESRD diabetics
Olmsted Cty 1994–03	9.0	1.8	2.1	0.7	1.0	257	43
Victoria 1995–97	12.9	2.2	4.3	1.8	1.3	101	35
Kurdistan 2012–13	5.1	1.6	0.4	0.7	1.2	60	23

Age standardized annual incidence adjusted to the 2000 US standard population

^a^ Incidence of ESRD per million population is unadjusted

Figure 1 demonstrates the changes in age specific incidence of all GN with increasing age in Kurdistan, Olmstead County, and Victoria. The plots show a plateau and then decline of age specific rates in Kurdistan at 40–45 years and moderately elevated rates with older age in Olmstead county that began to decline at approximately age 60. This contrasts with the marked elevation in incidence with age in Victoria with little indication of a late plateau. Although, the age standardized rates for FSGS and MGN were somewhat increased in Victoria compared to Kurdistan, the differences were largely the result of the late age of onset in Victoria and their infrequency in the elderly of Kurdistan.

### Incidence of end-stage renal disease in Kurdistan compared to the kingdom of Jordan

In 2017 in Sulaimaniyah, 79 patients initiated dialysis and 124 were repeatedly dialyzed for a total of 203 patients receiving dialysis and an initiation to total dialysis ratio of 0.39. A first time renal transplant was received by 46 Sulaimaniyah patients, but we could not identify who had been previously dialysed, and transplant patients were not included in our calculations of ESRD rates.

**Fig. 2:**
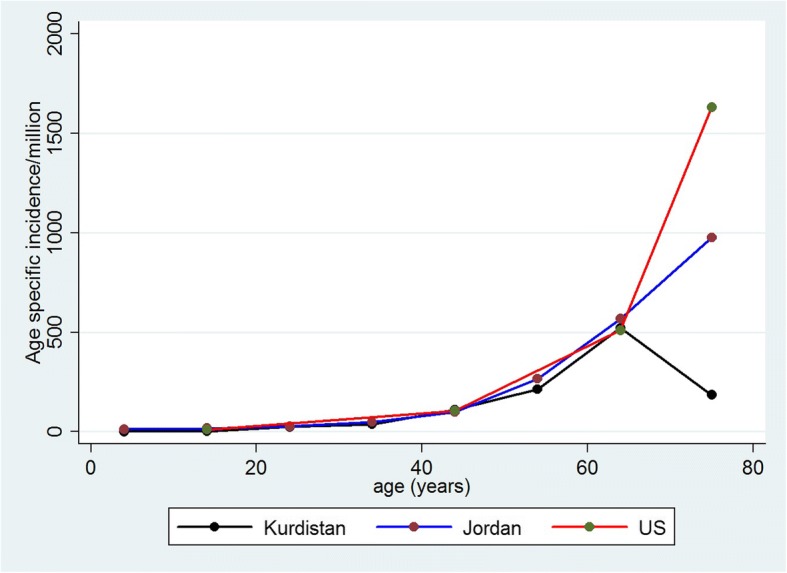
Age specific incidence of end-stage renal disease (ESRD) in Kurdistan, Jordan, and the United States (US. After approximately 55 years of age, there is a marked increase in age specific ESRD incidence in Jordan and the US. This is not seen in Kurdistan and is attributed to low rates of diabetic ESRD in the elderly (assigned cause of ESRD: Kurdistan, diabetes 23%, hypertension, 18%, glomerulonephritis, 12%; Jordan, diabetes 54%, hypertension, 26%, glomerulonephritis, 4%; US, diabetes 44%, hypertension, 28%, glomerulonephritis, 6%)

The age distribution and the age specific incidence (ASpI) of ESRD per million were as follows: 0–4 years old, 0 patients, ASpI = 0.0; 5–14 years old, 1 patients, ASpI = 3.0 per million; 15–24 years old, 7 patients, ASpI = 23.9 per million; 25–34 years old, 8 patients, ASpI = 36.4 per million; 35–44 years old, 11 patients, ASpI = 110.0 per million; 45–54 years old, 16 patients, ASpI = 210.5 per million; 65–64 years old, 24 patients, ASpI = 521.7 per million; ≥ 65 years old, 12 patients, ASI = 190.5 per million. The crude ESRD incidence was an estimated 60 persons per million. ESRD was attributed to diabetes in 23% of patients, hypertension in 18%, and glomerular disease in 12% as previously reported [20]. In Jordan, the age specific ESRD incidence at 55–64 years of age as well as in younger age groups was similar to that in the Kurdish region (Fig. 2). After age 65, the Jordanian rate doubled the 522 per million seen at the 55–64 years of age while the Kurdish regional rate declined. The crude incidence of Jordanian ESRD was 99 per million with 54% of ESRD ascribed to diabetes, 26% to hypertension, and 4% to glomerular disease [19]. Figure 2 graphs the changing age specific ESRD incidence for the Northern Iraqi and Jordanian populations and adds 2011 US rates for comparison [5, 19].

### Estimates of obesity in the Sulaimaniyah governate

This survey was conducted out-of-hospital within city neighborhoods. Clothed body mass index (BMI) ≥ 30 kg/m^2^ was the definition for obesity. Obesity was found among 4.1% of 125 males and 14% of 160 females. For persons over 50 years old, the obesity rate was 15% for men and 36% for women. In a sample of 108 children between 5 and 15 years old, 6 (5.6%) were classified as obese.

## Discussion

In 2013, Anand et el. [15] proposed using estimates of use and initiation of renal replacement therapy as a method of quantitating ESRD in developing countries. They pointed out that renal disease registries are non-existent in most of the world but emphasized the need for data on regional requirements for renal replacement therapy. Northern Iraq has 14 government dialysis centers with no conditions that exclude any person from receiving services. Daily and monthly records include name, age, blood pressures, and diabetes status. Date of entry into or exit from dialysis is rarely available, and data are not collected into a regional repository for formal estimates of incidence or prevalence.

We believe that rates of the initiation and the repeated use of dialysis when sampled at multiple dialysis centers can serve as surrogates for the incidence (initiation) and prevalence (total use, initiated and repeated) of ESRD. Our proportion of patients initiating dialysis in 2017 to the total number of patients using dialysis was 0.39. This is very close to the 0.34 reported by Anand et al. [15] for the Middle-East and North Africa as a whole and supports the validity of the concept as it is applied to our region. Using this approach, the annual crude incidence of ESRD for Iraqi Kurdistan was estimated at 60 per million. This is similar to an incidence of 64 per million reported in a 2014 review [21] from Iran that lies just to the East of Iraq but is considerably lower than Jordan that is on the Western Iraqi border [19].

Nevertheless, before 65 years old, age specific incidence rates for Jordan and Kurdistan were very similar to each other and to the US suggesting that before late middle age, ESRD rates in the Middle East resemble other geographic regions and that the major difference between low and high incidence regions is the accrual of CKD in aging populations. In this regard, it is important to recognize that Kurdistan and Jordan have young populations that contrast with the older population structures of the US and Australia. When standardized to the 2000 US population, the ESRD incidence for Kurdistan was still comparatively low at 124 per million; while the age standardized rate for Jordan was 237 per million, a value close to that for Olmstead County and twice as high as Australia [13, 22].

In Northern Iraq, FSGS was the most frequently diagnosed type of GN. FSGS had an age adjusted incidence of 1.6 per 100,000 that was close to the incidence of 1.8 per 100,000 seen in Olmstead County and was not markedly lower than the incidence of 2.2 per 100,000 in Victoria [13, 14]. Nevertheless, FSGS was diagnosed at a younger age in Kurdistan than in Olmstead County or Victoria. The incidence of FSGS and all GN in Kurdistan peaked at approximately age 40 and then began to decline; whereas, in Victoria and Olmstead County there was a continued rise in later years [13, 14].

The rates of diagnosis and the age of FSGS in a population raise at least two epidemiologic issues. One concerns the variable frequency of FSGS in different populations in which there is little relationship to the population risk for ESRD. This is in part because the major cause of ESRD in high incidence populations is diabetes. The US has the highest national incidence of ESRD in the world at a 2010 unadjusted rate of 369 per million with approximately 44% of new US ESRD patients being diabetic [4, 5]. In Australia, the rate of ESRD is considerably lower than the US at 101 per million, and just fewer than 35% of patients are diabetic [13]. IGAN is the most common form of GN in Australia, and contributes to nearly twice the frequency of GN-related ESRD as FSGS (IGAN 44% vs FSGS 23%) [13]. While these data do not indicate that FSGS or GN overall will add to a large future burden of ESRD in regions where diabetes is prevalent, the impact on lower risk regions is not known, and if FSGS in Iraq begins at an early age, a second issue is whether FSGS will increase in frequency as the young population ages.

In the US, FSGS was not always a commonly recognized disease, but it is currently the leading cause of nephrotic syndrome in both African Americans and whites [9, 23–27]. In the US, the risk of ESRD owing to FSGS is 4-times greater in African Americans, and African Americans are diagnosed with more severe disease at a peak age of 35 to 43 years old compared to 47 to 57 years old among whites [27, 28]. There is not any indication that age per se has any effect on prognosis, but FSGS has a generally poor out-come, and early versus late age of onset of any CKD results in a significantly increased loss of “kidney life” [29, 30].

FSGS appears to be increasing in many countries of the Middle-East and replacing SLE as the most common type of GN [31–33]. SLE is well known to have geographic, racial, and ethnic variations [13, 34, 35]. Some of the geographic differences are undoubtedly due to biopsy practices. The Australian biopsy rate is > 20 per 100,000. The US rate is approximately 17 per 100,000, less than Australia but higher than the 4–8 per 100,000 practiced by nephrologists in most parts of the world and the 7.8 per 100,000 in Kurdistan [13, 36]. High biopsy rates, particularly for minor abnormalities, will certainly increase rates of IGAN and less active forms of SLE.

The validity of the reported causes of ESRD in the US and other developed countries has been questioned [37]. A patient with long-standing diabetes will be appropriately considered to have diabetic nephropathy, but hypertension is problematic, as physician biases have been shown to influence the assignment of hypertensive nephropathy to ESRD patients rather than exploring other causes [37].

With biopsies, a diagnosis of a specific cause of late stage kidney disease can often be made [38]. End-stage glomerulonephritis is characterized by solidified glomeruli that in the case of immune-complex disease frequently contain immunoglobulin deposits, and in IGAN, IgA deposits usually remain with advanced glomerulosclerosis [38]. Hypertension progresses by increasingly severe arteriosclerosis and glomerular loss that is primarily the result of ischemic glomerular obsolescence [39]. A predominance of glomerular solidification with hyalinosis lesions and IgM and C3 deposits favors primary FSGS as a cause of ESRD [39, 40]. Our analysis of causes of biopsy determined ESRD in Iraqi patients indicates that the proportional contribution of FSGS to non-diabetic ESRD could be as high as 41% and suggests that FSGS could raise ESRD rates if the disease followed the patterns in the US and Australia and became more common in older members of the population.

One hypotheses for the lowered rates of ESRD and FSGS in the elderly of Northern Iraq is that physicians stop looking for disease in older patients, or older persons become oblivious to their illnesses and do not seek medical help. We believe that these are unlikely explanations. The elderly are valued members of Iraqi and Kurdish society, and there is no age discrimination for any form of medical service.

A second hypothesis is an age cohort effect in which older members of the population have not been exposed to the factors causing the disease. This certainly could affect rates of diabetes and ESRD, as older Iraqis have been living in deprived conditions imposed by international trade restrictions since the 1980s. A current 26–30% rate of obesity is reported for Southern Iraq [41, 42], but this is excessive for Sulaimaniyah, where the all-age obesity rate was less than 10%. The obesity rate of 36% in Jordan [41] may portend changes that will be seen in the future, but we do not see a large segment of the Kurdish population currently at risk for diabetic kidney disease.

Although both Iraq and Jordan have similarly young populations, the estimated risk of ESRD in Northern Iraq was low; while the Jordanian population when adjusted for age had a risk of ESRD that more resembled the US. These differences highlight the impact of undernutrition and over nutrition on kidney health. Political instability is an unfortunate fact-of-life in many parts of the Middle-East and North Africa. Somalia and Sudan are examples where war and continuous undernutrition have lead to endemically high rates of hypertension and non-diabetic CKD possibly influenced by an intra-uterine derived nephron deficit [43, 44]. Diabetes as a disease of overnutrition and ageing will almost certainly become more common as economies improve. FSGS appears to be increasing throughout the Middle-East, and we suggest that GN and particularly FSGS are also age-dependent with effects on ESRD that are unlikely to be known for many years.

## Conclusions

At an annual rate of 60 per million, the incidence of ESRD in Northern Iraq is much lower than the crude incidences of 100 and 390 per million for Jordan and the US respectively. This difference is primarily the result of low identified rates of renal disease in the Iraqi elderly and a relative infrequency of diabetes. With the exception of IGAN, that has a low incidence in the region, rates of the major types of glomerular diseases are similar to the West. FSGS is the most common primary glomerular disease in Northern Iraq. It has an age adjusted annual incidence of 1.6 cases per 100,000 population and makes an apparent major contribution to regional ESRD.

## Abbreviations

ASI: Age standardized incidence
ASpI: Age specific incidence
CGN-NOS: End-stage chronic GN not otherwise specified
CKD: Chronic kidney disease
ESKD: End-stage kidney disease
ESRD: End-stage renal disease
FSGS: Focal segmental glomerulosclerosis
GN: Glomerulonephritis
HUS: Hemolytic uremic syndrome
IGAN: Immunoglobulin A nephropathy
MCD: Minimal change disease
MGN: Membranous GN
MPGN: Membranoproliferative GN
SLE: Lupus nephritis
TIN: Tubulointerstitial nephritis
US: United States
